# A rare case of gouty arthropathy in the spine complicated with acute cord compression

**DOI:** 10.1259/bjrcr.20220048

**Published:** 2022-05-13

**Authors:** Brian Fung, WH Chong, Chun Hung Kevin Yu, Siyue Yang, Cheuk Him Ho, Li On Chee Angela

**Affiliations:** 1 Tuen Mun Hospital Hong Kong, New Territories, Hong Kong

## Abstract

Gout is one of the most common inflammatory arthropathies in the developed world. However, involvement of the spine is relatively rare, and other sinister differential diagnoses will need to be considered. We describe an unusual case of gouty tophi deposition within the spine in an elderly patient presenting with signs and symptoms of acute cord compression. Important differential diagnoses that need to be excluded include bony metastases from underlying malignancy and other infective/inflammatory causes. Early recognition of imaging findings can avoid delayed or inappropriate medical treatment.

## Clinical presentation

A 70-year-old male with longstanding history of multiple joint pain and soft tissue swelling, and multiple other co-morbidities including diabetes, hypertension and chronic renal failure, presents with sudden onset of upper and lower limb weakness. Physical examination was worrisome for spinal cord compression.^1,2^

## Imaging findings

**Figure 1.**
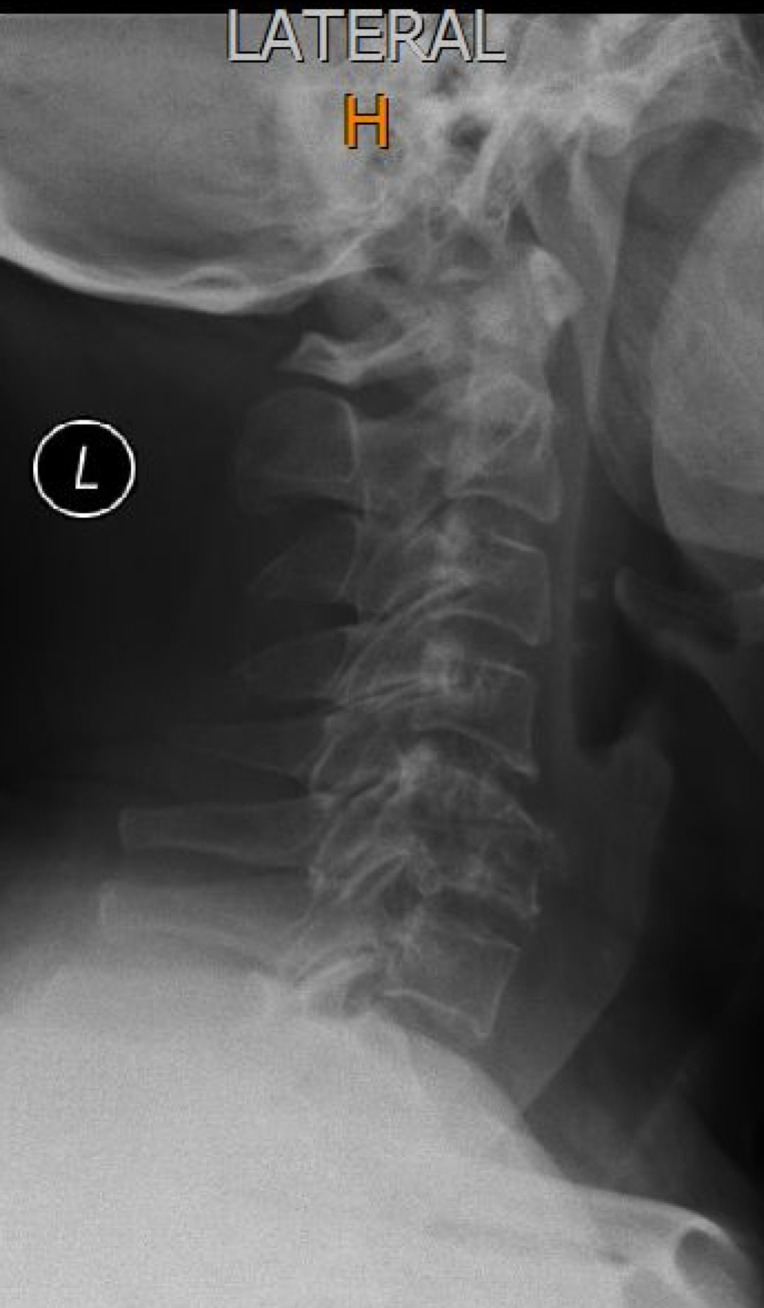
Lateral cervical spine radiograph showing partial collapse of the C5 and C6 vertebral bodies with complete loss of the C5/6 disc space

A lateral view of the cervical spine showed partial collapse of the C5 and C6 vertebral bodies with complete loss of the C5/6 disc space (Figure 1).

**Figure 2.**
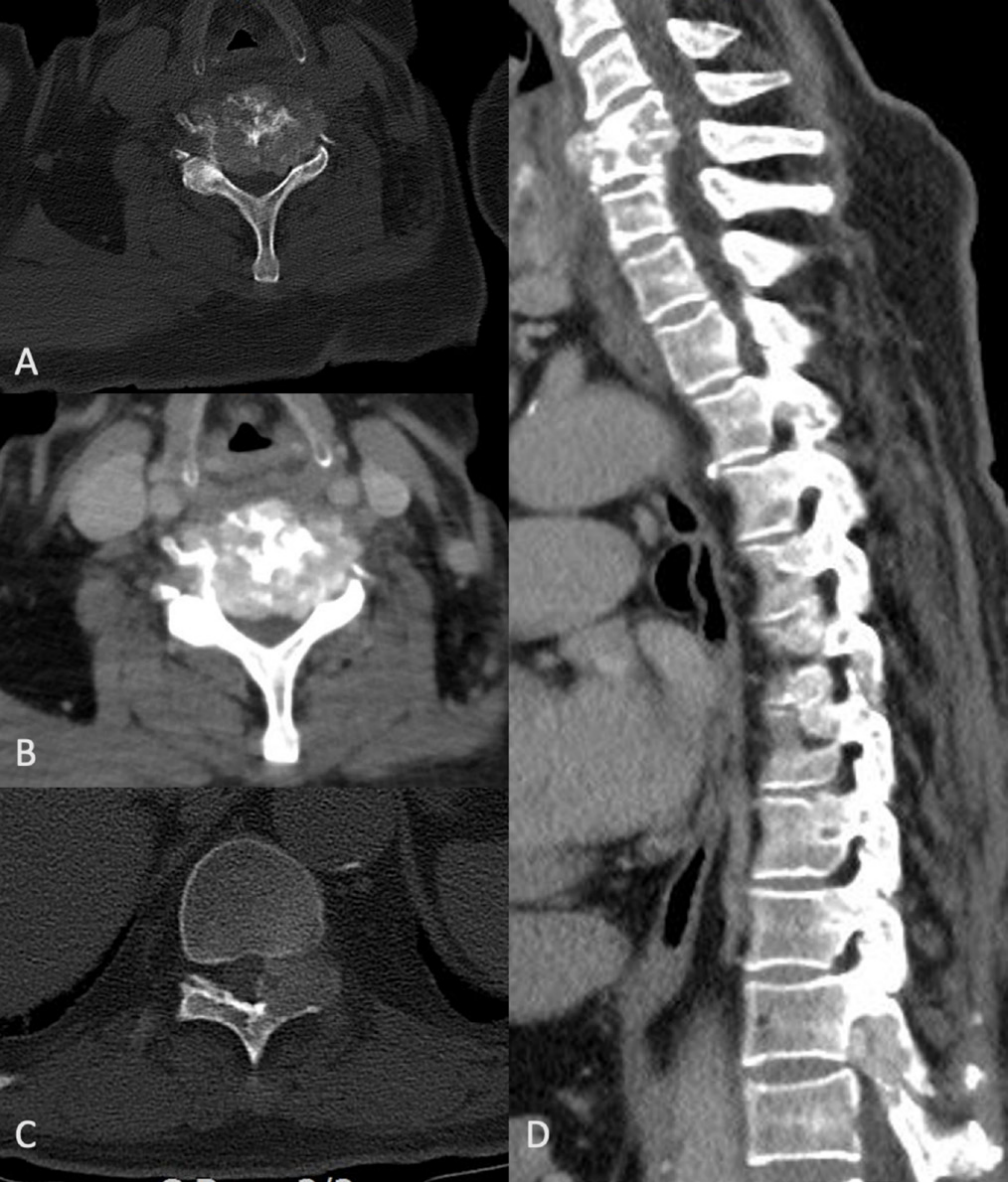
A, axial bone window CT image showing bulging soft tissue mass causing narrowing of spinal canal and obliteration of bilateral neural foramina at the C5 level; B, post contrast CT image showing avid enhancement of the soft tissue; C, another soft tissue lesion at the left pedicle of T10 vertebra causing narrowing of the spinal canal; D, sagittal reformatted images showing multi-level involvement

Contrast-enhanced computed tomography (CT) of the cervical and thoracic spine was performed with images obtained in pre-contrast, post contrast and bone window to further delineate the pathology. On the axial images in bone window, a bulging soft tissue mass causing narrowing of spinal canal and obliteration of bilateral neural foramina at the C5 level Figure 2a with avid contrast enhancementis Figure 2b seen. Another soft tissue lesion with similar imaging appearance was seen at the left pedicle of T10 vertebra, causing narrowing of the spinal canal Figure 2c. Sagittal reformatted images showing extent of soft tissue deposits Figure 2d. These CT findings were worrisome for metastatic lesions, and further evaluation with magnetic resonance imaging was performed.

**Figure 3.**
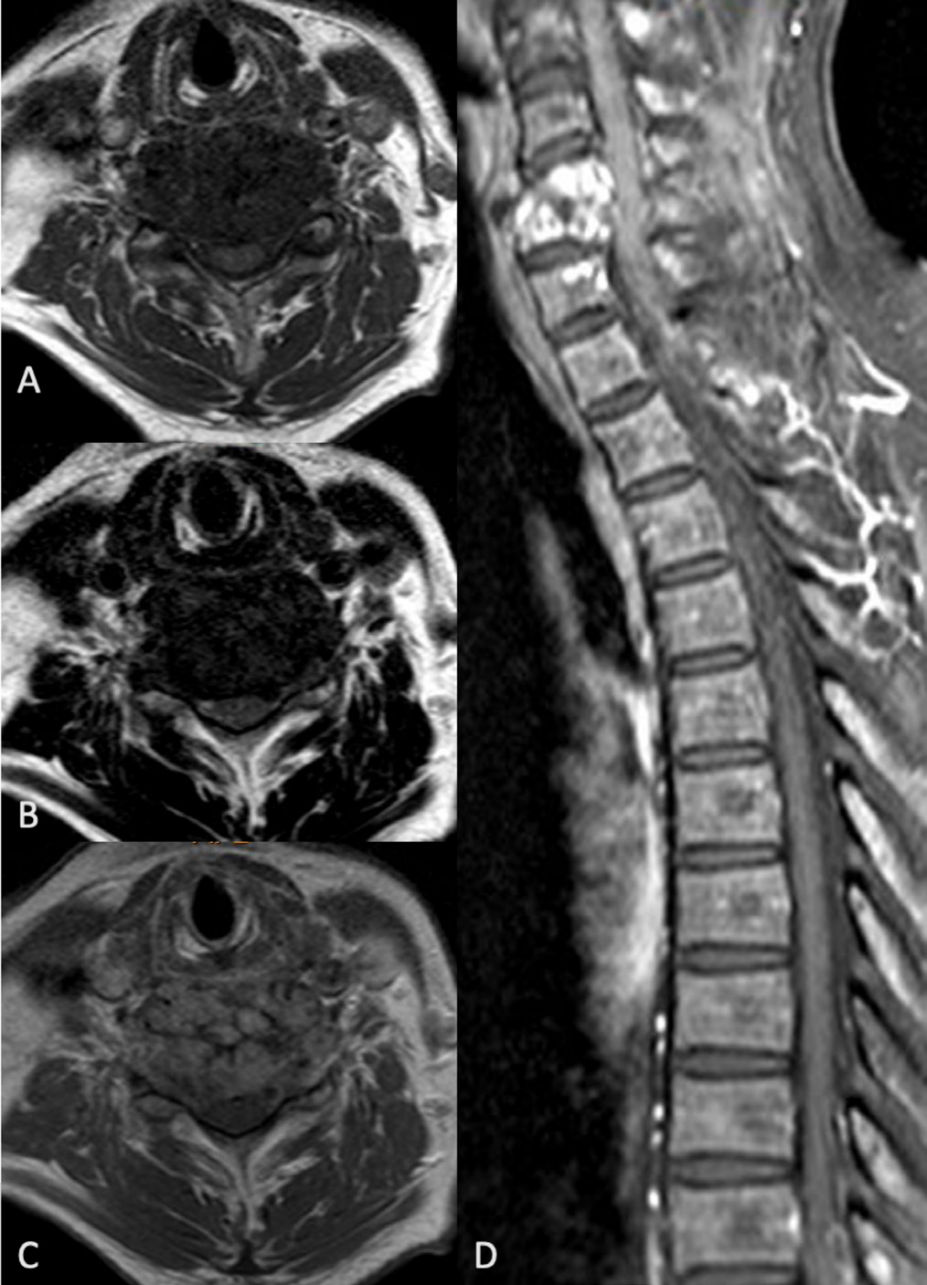
A, B,*T*_1_-weighted and *T*_2_-weighted MR images showing intermediate signal lesion centered at the intervening disc space intermediate signal lesion centered at the intervening disc space; C, heterogenous contrast enhancement with apparent bony expansion; D, sagittal MR images showing compression onto the cervical cord

**Figure 4.**
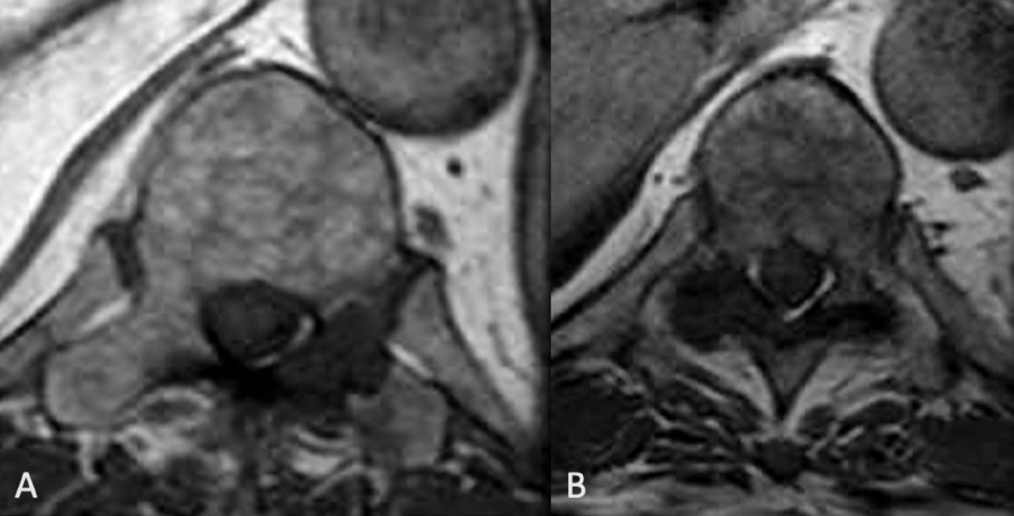
A, B, Other lesions seen at para-articular or juxta-articular location to the facet joints

Magnetic resonance imaging of the whole spine with contrast showed collapse of C5 and C6 vertebral bodies with a *T*_1_-weighted and *T*_2_-weighted intermediate signal lesion centered at the intervening disc space (Figure 3a, b). There was heterogenous contrast enhancement and apparent bony expansion (Figure 3c) and sagittal MR images showing compression onto the cervical cord (Figure 3d). Bilateral C5/C6 intervertebral foramina were obliterated with extradural compression on thecal sac and effacement of anterior subarachnoid space. Multiple other lesions with similar signal intensities were also observed affecting the posterior elements of spine at multiple levels, some of which were in a para-articular or juxta-articular location to the facet joints (Figure 4a, b).

The presence of para-articular and juxta-articular disease involvement around the facet joints is not typical of metastasis nor infection. In this instance, depositional disease, in particular gout, is a consideration.

^99m^Tc bone scintigraphy was then performed, demonstrating background polyarthritis, involving bilateral feet and knees. Abnormal tracer uptake was noted at C5/6 level. There were also multiple foci of elevated tracer uptake in the posterior elements of the thoracic spine, which is atypical for osseous metastases.

## Differential diagnosis

Important differential diagnoses that need to be excluded includes bony metastases from underlying malignancy, and infections such as bacterial or tuberculosis. Calcium pyrophosphate deposition disease (CPPD) or pseudogout, can present in a similar fashion as gout, but can be differentiated radiologically by the presence of chondrocalcinosis commonly in the hands, wrist or knees. CPPD also seldomly has a soft tissue component as seen in gout^3^

### Outcome and treatment

A CT-guided bone biopsy of T10 and T11 vertebral bodies was performed and histology showed foreign body granulomatous reaction towards eosinophilic fibrillary material, suggestive of tophaceous gout. Patient was referred to the rheumatologist and neurosurgeon for further management during this in-patient stay, but unfortunately succumbed to hospital acquired pneumonia before further treatment for his gout and cord compression could be administered.

## Discussion

Gouty arthropathy is an asymmetrical polyarticular disease, with the foot being the most common in location and in particular the first metatarsophalangeal joint. The next most common regions are the hands and wrists, followed by the elbow and knee joints.^3^

There can be involvement of any segment of the spine, including the vertebral bodies, pedicles, lamina, ligaments, epidural, and intradural spaces. Lumbar spinal involvement is most commonly affected in the spine.^8^ Michael et al has reported that most cases (80%) involved only one region of the spine, and around 25% affected the cervical spine only.^9^ To our knowledge, only around 10 or so cervical gout cases with images have been reported so far.

Gouty tophi may vary considerably in radiological manifestation, depending on their histological composition. They commonly appear as round or oval ill-defined hyperdense mass lesions in comparison with normal soft tissue. Small lesions can also be radiographically occult if less than 10 mm in size.^4^ Tophi are predominantly juxta-articular in location. Occasionally, it may arise from within the bone, giving rise to lytic, expansile, well-defined lesions with thin sclerotic margins.^5^

Dual-energy computed tomography (DECT) is an increasingly common investigation for evaluating tophaceous gout deposition.^1^ The principle and value of DECT is its ability to differentiate densities based on their relative absorption of X-rays at different photon energy levels (commonly at 80 and 140 kVp),^6^ which enables quantification of gouty deposits and demonstration of non-crystalline tophus components with greater resolution.^5^

Gouty tophi on MRI typically shows *T*_1_-weighted intermediate signal and homogeneous enhancement post-contrast. Differences in its calcium concentration can give rise to variable and *T*_2_-weighted signal. Bone weakening can lead to pathological fractures and locoregional mass effect/compression by the tophi, as demonstrated in this case^1^

Histological diagnosis is confirmed with microscopic monosodium urate crystals in synovial fluid or within the tophi.^7^ Disease presentation in atypical locations may warrant a bone or soft tissue biopsy to exclude other sinister causes.

Conservative medical treatment can be considered in patients without concomitant neurological deficits, after excluding spinal infection or early reversible causes. Nonsteroidal antiinflammatory agents and steroids have been proven to be effective for acute pain control. Surgery may be considered when there is a need for relief of neurologic symptoms, but long-term management still relies on adequate medical therapy.^7^

## Learning points

- This case illustrates a common disease entity presenting in an atypical location and presentation with complications.
- Involvement of the vertebral column is uncommon in gouty arthropathy, and causing acute cord compression is even rarer. Important common differential diagnoses that needs to be excluded are bony metastases from underlying malignancy, and other infective/inflammatory changes which warrant more urgent management.
